# Glycomacropeptide Reduces Intestinal Epithelial Cell Barrier Dysfunction and Adhesion of Entero-Hemorrhagic and Entero-Pathogenic *Escherichia coli* in Vitro

**DOI:** 10.3390/foods6110093

**Published:** 2017-10-27

**Authors:** Shane Feeney, Joseph Thomas Ryan, Michelle Kilcoyne, Lokesh Joshi, Rita Hickey

**Affiliations:** 1 Teagasc Food Research Centre, Moorepark, Fermoy, P61C996 Co. Cork, Ireland; shanefeeney1518@gmail.com (S.F.); josephthomas.ryan@gmail.com (J.T.R.); 2 Advanced Glycoscience Research Cluster, National Centre for Biomedical Engineering Science, National University of Ireland Galway, H91TK33 Galway, Ireland; michelle.kilcoyne@nuigalway.ie (M.K.); lokesh.joshi@nuigalway.ie (L.J.)

**Keywords:** *Escherichia coli*, adherence, enterohemorrhagic, enteropathogenic, glycomacropeptide, milk

## Abstract

In recent years, the potential of glycosylated food components to positively influence health has received considerable attention. Milk is a rich source of biologically active glycoconjugates which are associated with antimicrobial, immunomodulatory, anti-adhesion, anti-inflammatory and prebiotic properties. Glycomacropeptide (GMP) is the C-terminal portion of kappa-casein that is released from whey during cheese-making by the action of chymosin. Many of the biological properties associated with GMP, such as anti-adhesion, have been linked with the carbohydrate portion of the protein. In this study, we investigated the ability of GMP to inhibit the adhesion of a variety of pathogenic *Escherichia coli* strains to HT-29 and Caco-2 intestinal cell lines, given the importance of *E. coli* in causing bacterial gastroenteritis. GMP significantly reduced pathogen adhesion, albeit with a high degree of species specificity toward enteropathogenic *E. coli* (EPEC) strains O125:H32 and O111:H2 and enterohemorrhagic *E. coli* (EHEC) strain 12900 O157:H7. The anti-adhesive effect resulted from the interaction of GMP with the *E. coli* cells and was also dependent on GMP concentration. Pre-incubation of intestinal Caco-2 cells with GMP reduced pathogen translocation as represented by a decrease in transepithelial electrical resistance (TEER). Thus, GMP is an effective in-vitro inhibitor of adhesion and epithelial injury caused by *E. coli* and may have potential as a biofunctional ingredient in foods to improve gastrointestinal health.

## 1. Introduction

Prevention and treatment of infectious diseases requires a thorough understanding of the complex interactions between pathogenic bacteria and the human host. It is estimated that at least 90% of all bacteria in the environment survive attached to or in close association with a surface, where they can thrive [1]. Bacterial colonisation and infection of the gastrointestinal tract involves the binding of bacterial adhesins to specific ligands present on the intestinal epithelium [2]. Bacterial survival then increases after this attachment is made, as bacteria are more resistant to cleansing mechanisms, immune factors, bacteriolytic enzymes, antibiotics and physical removal by hydrodynamic forces [3]. Hence, early prevention of bacterial adherence to the host epithelium should reduce the incidence of disease.

Pathogenic *Escherichia coli* is one of the leading causes of intestinal (enteritis, diarrhea, or dysentery) disease. When *E. coli* adheres to cells lining the intestine, disruption of normal intestinal barrier function occurs. This can result in the leakage of water and plasma proteins into the lumen and translocation of intestinal bacteria into the systemic circulation, contributing to the development of systemic septicaemia [4,5]. After adhesion, enteropathogenic *E. coli* (EPEC) and enterohemorrhagic *E. coli* (EHEC) insert bacterial effector proteins and translocated intimin receptor (TIR) into the host cell via the type III secretion system (TTSS), which are known to cause gut barrier dysfunction leading to increased cell permeability through recruitment of pro-inflammatory cytokines such as interleukin 8 (IL-8) and tumor necrosis factor alpha (TNF-α) [6,7,8,9,10,11,12]. These inflammatory cytokines modulate intracellular signalling pathways within the host that promote and redistribute tight junction (TJ) proteins such as zonula occludens-1 and claudin, which increases membrane permeability and paracellular movement of bacteria [13].

Previous studies have suggested that treatment with certain glycoproteins could help prevent considerable structural and functional damage caused by inflammation [12,14,15]. Milk glycoproteins have also been shown to obstruct specific host–pathogen interactions including bacterial adhesion to the host ligands [16,17,18]. Glycomacropeptide (GMP) is a casein-derived whey protein found in “sweet” whey, and is formed when kappa-casein is hydrolysed by chymosin during cheese production [19]. Previously, it has been reported that GMP can inhibit viral or bacterial adhesion to cells [20,21], promote proliferation of beneficial bacteria [22,23], neutralize enterotoxin, inhibit gastrointestinal secretions and exert immune regulation [24]. These bioactivities are mainly attributed to the O-linked glycosylation [25] associated with GMP and particularly the sialic acid (*N*-acetylneuraminic acid) component (reviewed by [26]). Sialic acid is also present on surface receptors of intestinal cells and has been identified as a ligand component for bacterial adhesion [27,28,29].

In previous studies, GMP has been shown to reduce the adherence of pathogens such as *Salmonella enteritidis*, *S. fyris*, *S. typhimurium*, *Vibro cholera*, *Helico pylori*, *Shigella flexneri* and *E. coli* to certain intestinal cell lines [2,16,20,21,30,31]. Nakajima et al. [30] found that GMP could inhibit the association of EHEC O157 with Caco-2 cells and it has also been shown to inhibit the association of EPEC with Caco-2 cells based on pathogen-binding to its sialic acid component [20]. The glycopeptide was also found to inhibit the adhesion of certain strains of EPEC to human HT29 cells [2] and the ETEC strain K88 to porcine intestinal cells and porcine mucus [32,33].

In this study, we aimed to directly compare the ability of GMP to inhibit adhesion of EPEC and EHEC strains to two intestinal cell lines to determine any bias of GMP towards inhibition of certain pathotypes or when using different cell lines. The study also aimed to establish the mechanism of anti-adhesion, whether it be through either direct (bacterial binding) or indirect (cell-line binding) inhibition, and to assess the ability of GMP to suppress pathogen-induced tight junction (TJ) barrier function impairment.

## 2. Materials and Methods

### 2.1. Materials

GMP containing approximately 8.5% sialic acid on a GMP basis was kindly provided by Agropur Ingredients (Eden Prairie, MN, USA). The human colonic adenocarcinoma cell lines, HT-29 and Caco-2, were purchased from the American Type culture collection (ATCC). Cell culture reagents were purchased from Sigma-Aldrich (Wicklow, Ireland).

### 2.2. Bacteria and Culture Conditions

The EPEC strains O111:H19 (NCTC 8007) and O125:H2 (NCTC8623) and EHEC strains NCTC12900, DAF 454 and DPC 6055 were obtained from the Leibniz Institute DSMZ-German Collection of Microorganisms and Cell Cultures (DSM; Braunschweig, Germany), the National Collection of Type Cultures (NCTC; London, UK) and the Dairy Products Research Centre culture collection (DPC; Teagasc Food Research Centre, Cork, Ireland). Strains were stocked in brain heart infusion (BHI) broth (Oxoid^®^ Ltd., Basingstoke, Hampshire, England) containing 50% glycerol (*v*/*v*) and stored at −20 °C. All strains were cultured directly from storage into BHI broth and incubated under aerobic conditions at 37 °C.

### 2.3. Mammalian Cell Culture

HT-29 and Caco-2 cells were routinely grown in McCoy’s 5A modified medium and Dulbecco’s modified eagle medium (DMEM), respectively, both supplemented with 10% fetal bovine serum (FBS). All cells were maintained in 75 cm^2^ tissue-culture flasks and incubated at 37 °C in 5% (*v*/*v*) CO_2_ in a humidified atmosphere. Cells were passaged when the confluency of the flask was approximately 90% as previously described [34]. Cells were trypsinized and seeded onto 12-well PVDF (Polyvinylidene fluoride) membrane plates (Corning) at a density of 1 × 10^5^ cells/well as previously described [34]. For translocation studies, Caco-2 cells were seeded onto transwell inserts (0.4 μm pore size, 12 mm diameter) (Corning), where they formed a tight monolayer. The media was changed every other day and the cells were grown until they reached confluence (1000 Ω) after 17–20 days.

### 2.4. Adhesion Assay

A series of adhesion assays, adapted from [35,36], were performed with HT-29 and Caco-2 cells and *E. coli* in the absence (control) and presence of GMP resuspended in either McCoy’s 5A modified medium or DMEM, respectively. Cells were cultured as above for 48 h and the media was supplemented with 2% FBS 24 h prior to inhibition studies. *E. coli* were harvested from the BHI, washed three times in phosphate-buffered saline, pH 7.2 (PBS), and diluted to an OD_600nm_ of 0.2 (approximately 1 × 10^8^ colony forming units (CFU)/mL) in either McCoy’s 5A modified medium or DMEM. Prior to infecting the cell line, *E. coli* was pre-incubated with filter-sterilised GMP (5 mg/mL), for 1 h at 37 °C. The adhesion assays were conducted on three separate occasions in triplicate.

#### 2.4.1. Standard Inhibition Assay

Confluent monolayers of HT-29 cells were washed twice in PBS and infected with *E. coli* (1 × 10^8^ CFU/mL) which had been pre-incubated with 5 mg/mL of GMP or the respective buffer (control) for 1 h at 37 °C (5% CO_2_). The cells were then incubated for 1 h at 37 °C and 5% (*v*/*v*) CO_2_. To determine the number of cell-associated bacteria, each well was washed five times with PBS and the cells were lysed with 500 μL of 0.1% Trition X-100 in PBS for 30 min. Serial dilutions of the cell lysates were then plated onto BHI agar and incubated for 12 h at 37 °C, after which CFU/mL was calculated.

#### 2.4.2. Concentration Dependency Assay

Prior to infecting the HT-29 cell line, *E. coli* NCTC12900 (1 × 10^8^ CFU/mL) was pre-incubated for 1 h at 37 °C (5% CO_2_) with various concentrations of GMP (1, 2, 3, 4, 5 and 6 mg/mL) in McCoy’s 5A media (2% FBS). After this step, the inhibition assay was performed as described above.

#### 2.4.3. Bacterial Interaction with GMP

Prior to infecting the HT-29 or Caco-2 cell lines, *E. coli* NCTC12900 (1 × 10^8^ CFU/mL) was pre-incubated for 1 h at 37 °C (5% CO_2_) with GMP (5 mg/mL) in McCoy’s 5A media with 2% FBS. The samples were centrifuged at 4700 rpm for 7 min to pellet the bacterial cells. Media containing unbound GMP was removed and the bacterial pellet was then re-suspended in McCoy’s 5A media with 2% FBS. After this step, the inhibition assay was performed as described above.

#### 2.4.4. Cell-Line Interaction with GMP

The confluent monolayer was washed twice in PBS and 500 μL of the GMP (5 mg/mL) in McCoy’s 5A media with 2% FBS was added to the wells. Unbound GMP was removed by washing the mammalian cells five times in PBS prior to infection with non-pre-incubated bacteria and incubated for 1 h at 37 °C (5% CO_2_). The inhibition assay was then continued as described above.

#### 2.4.5. Instantaneous Effect of GMP

The confluent monolayer was washed twice in PBS and was simultaneously exposed to 500 μL of GMP (5 mg/mL) and non-pre-incubated bacteria and incubated for 1 h at 37 °C (5% CO_2_). The inhibition assay was then continued as described above.

### 2.5. Translocation and Transepithelial Electrical Resistance (TEER) Analysis

To investigate the effect of GMP on *E. coli* translocation, Caco-2 cells were grown on transwell inserts for 21 days in DMEM containing 10% (*v*/*v*) FBS, 1% (*v*/*v*) non-essential amino acids and 0.5% penicillin–streptomycin (5000 U/mL). Prior to infection, *E. coli* NCTC12900 (1 × 10^8^ CFU/mL) was pre-incubated with GMP (5 mg/mL) for 1 h at 37 °C before being applied to the apical side of the transwell plate. Serum-free media (1 mL) were added to the basolateral chamber and the cells were incubated for 14 h at 37 °C (5% CO_2_). Transwell inserts containing cells under the same conditions were also established for the introduction of non-treated bacteria in serum-free media (negative control). The number of translocating bacteria was determined by plating the basolateral medium onto BHI agar at 1, 2, 3, 4, 6, 8, 12 h post-apical infection as above. To confirm the formation of a tight cell monolayer, TEER measurements were carried out at 7, 14 and 21 days using an EVOM X meter coupled to an STX2 manual electrode (World Precision Instruments, Sarasota, FL, USA). To control for any disruption in the integrity of the cell monolayer during bacterial infection, TEER reads were taken at 1, 2, 3, 4, 6, 8, 12 h post-apical infection.

### 2.6. Statistical Analysis

Results from the inhibition and translocation studies are presented as mean ± standard deviations of replicate experiments. Statistical significance was determined using the unpaired student *t*-test and *p* < 0.05 was considered significant.

## 3. Results and Discussion

### 3.1. Anti-Adhesive Activity of GMP

**Figure 1:**
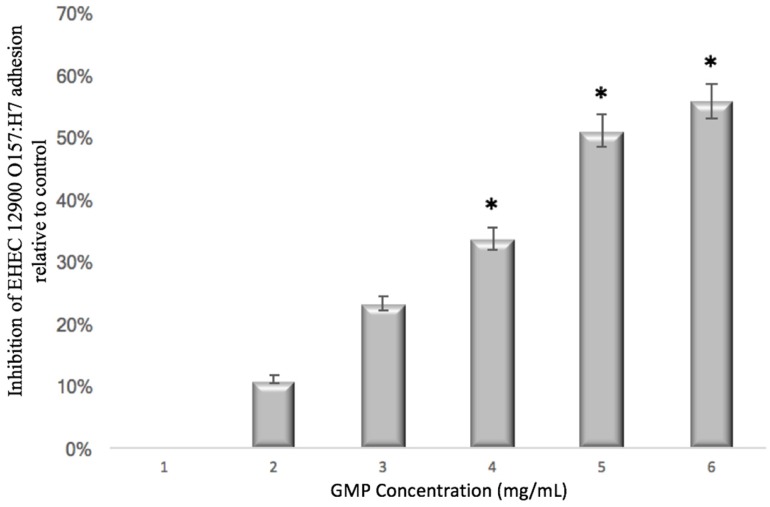
The effect of GMP concentration on inhibition of adhesion of EHEC 12900 O157:H7 to HT-29 cells (* *p* < 0.05). EHEC = enterohemorrhagic *E. coli*; GMP = Glycomacropeptide.

The ability of GMP to prevent pathogen adhesion was assessed using both HT-29 and Caco-2 cell lines (Table 1). Concentration dependency assays were performed to determine if inhibition was dependent on GMP concentration (Figure 1). EHEC 12900 adhesion was selected for this purpose and a concentration of 5 mg/mL was chosen for all studies thereafter. The range of concentrations used in this assay were selected given that 1.2–1.5 mg/mL of GMP is found in whey from cheese manufacturing [37]. Prior to mammalian cell infection, *E. coli* strains were pre-incubated with GMP. Subsequently, GMP was shown not to kill the bacteria, did not influence bacterial growth over the course of the assay and did not affect the viability of the HT-29 cells as confirmed by real-time analysis of cell viability using an xCELLigence system (Roche).

The greatest anti-adhesive activity in response to GMP was observed against EHEC 12900 (*p* < 0.001) with 70% and 62% inhibition of adhesion to HT-29 and Caco-2 cells, respectively, relative to the control (Table 1). Similarly, EPEC 0111:H2 (*p* < 0.05) and EPEC 0125:H32 (*p* < 0.05) adhesion to HT-29 and Caco-2 cells was also reduced in the presence of GMP by 26% and 24%, and 25% and 25%, respectively, relative to the control (Table 1). Inhibition of adhesion to HT-29 cells was observed for the other EHEC strains tested, but was not statistically significant. These results suggest that the effect of GMP on *E. coli* adhesion was not associated with a particular pathotype but instead appears to be strain specific. Interestingly, similar inhibition of the same strains by GMP was observed for both the Caco-2 and HT-29 cell lines, which indicates that the effect is not cell-line associated but instead may be strain specific (Table 1).

**Figure 2:**
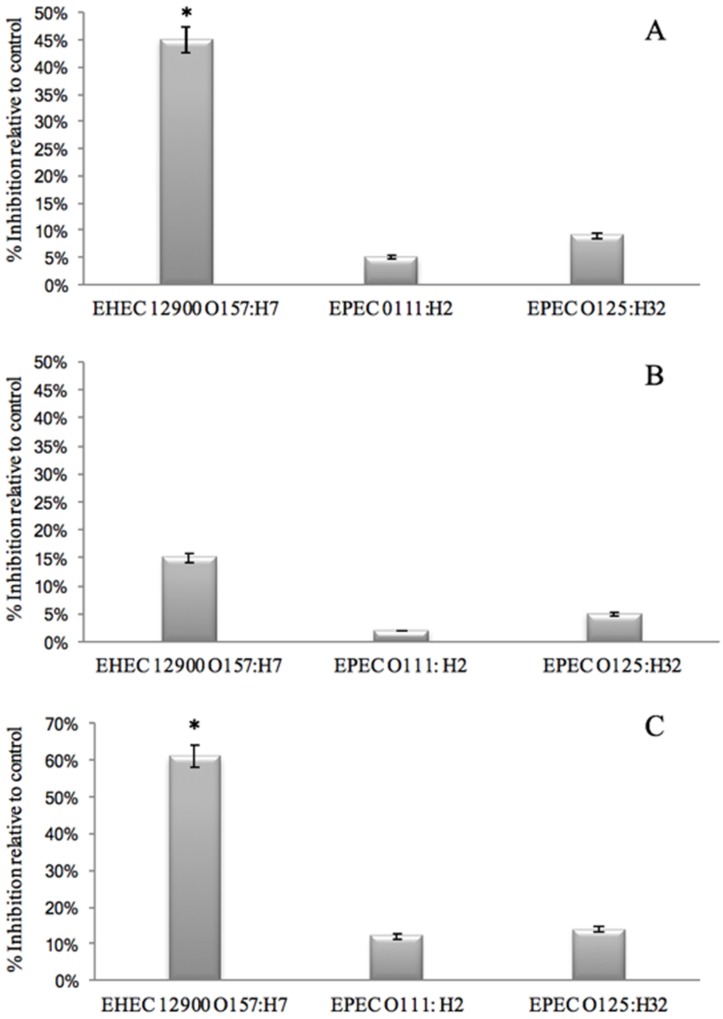
The effect of GMP on the association of *E. coli* strains with HT-29 cells. (**A**) Effect of pre-incubation of bacteria with the GMP for 1 h. (**B**) Effect of pre-incubation of HT29 cells with GMP prior to bacterial infection. (**C**) Effect of no pre-incubation step on anti-adherence effect of GMP (* *p* < 0.05). EHEC = enterohemorrhagic *E. coli*; EPEC = enteropathogenic *E. coli*.

Previous studies [2,20,21,30] indicated that GMP inhibited adhesion of *E. coli* to mammalian cells by interacting with the bacteria, thereby preventing human-cell association. However, GMP may also interact with the cell line to prevent bacterial interaction. Therefore, three different possibilities were assessed:

(1) That GMP interacts with bacterial binding sites, thereby preventing the bacteria from binding to HT29 cell receptors. This was investigated by removing unbound GMP from bacterial GMP mixture using centrifugation prior to infection. Anti-adhesive activity was still observed against EHEC 12900 adhesion to HT29 cells (*p* < 0.05). However, no significant anti-adhesive activity was evident against EPEC O111 and O125 (Figure 2A).
(2) That GMP binds to epithelial cell receptors, thereby preventing bacteria from interacting with the host cell surface. This was investigated by pre-incubating GMP with the cell line and washing off unbound GMP prior to bacterial challenge. No inhibition was observed against either EHEC or EPEC strains. These results suggest that GMP interacts with the bacteria but not the mammalian cells. It is interesting to note that removing unbound GMP from the bacteria reduced the anti-adhesive activity compared to co-incubating GMP with the bacterial and mammalian cells (Figure 2B).
(3) That pre-incubation of the bacteria with GMP is not required and that inhibition of adhesion can occur with simultaneous exposure of GMP and bacteria to the cell lines as would be more realistic of the in-vivo situation. No anti-infective activity was observed against the EPEC strains; however, an instantaneous anti-infective activity against EHEC 12900 (*p* < 0.05) was evident (Figure 2C) at levels comparable to using a pre-incubation step (Figure 2A). These results suggest that GMP does not require pre-incubation with the bacteria to exert its maximal inhibitory effect on *E. coli* cellular association, and a reduction in binding was evident instantaneously. No significant inhibition of adhesion of any of the strains was observed when using the Caco-2 cells in the three experiments outlined above.

It is known that *E. coli* adhere to epithelial cells of the intestine using adhesins such as pili and fimbriae, as well as capsular material found on the bacterial cells’ surfaces, and that these receptors adhere to specific glycosylated ligands found on intestinal epithelial cell surfaces [38,39]. Sialic acids and mannose have been found to be important specific host-cell receptors for the initial phase of infection (adhesion of lectins on the surface of bacteria to specific receptors on host cell [3,39,40,41,42]). Therefore, the current study and previous studies [30,43] further confirm that the bovine milk glycopeptide GMP, which contains multiple sialic acid residues, may inhibit the colonisation of intestinal cell lines by mimicking receptor sites on eukaryotic cell membranes, thereby blocking bacterial infection. Rhoades et al. [2] demonstrated that GMP did not significantly inhibit the adhesion of EPEC O111:H27 to ileal mucosa tissues obtained from piglets. However, in the present study, EPEC O111:H2 adhesion was significantly decreased. Therefore, the difference in anti-adhesive activity of GMP amongst *E. coli* strains could in part be flagella mediated. The results from the current study and previous studies suggest that GMP interacts with the bacterial surface and not the mammalian cell surface. Further studies are required in order to determine if GMP is blocking the bacteria and stopping cellular interactions solely as a mode of action, or if bacterial cell surface changes are occurring also in its presence (e.g., upregulation or downregulation of adhesin expression).

### 3.2. Effect of GMP on Caco-2 Tight Junction Integrity

**Figure 3:**
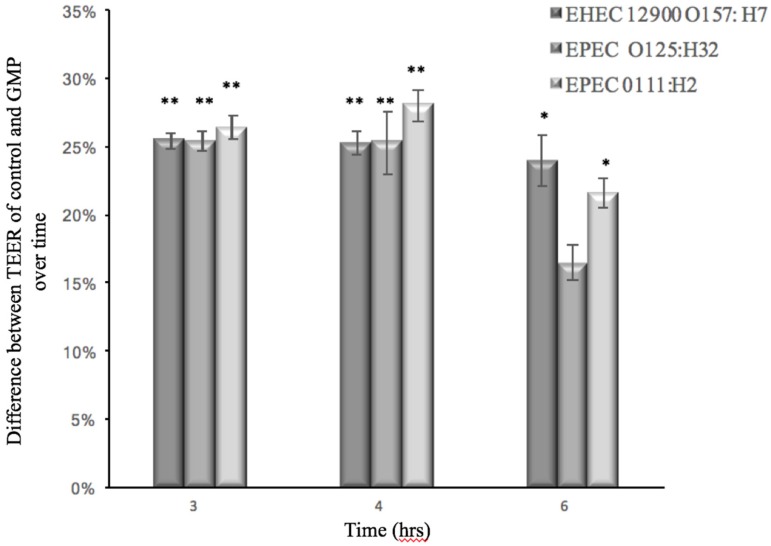
Percentage of prevention of reduction in Transepithelial Electrical Resistance (TEER) values of Caco-2 monolayers after infection with *E. coli* strains pretreated with GMP in comparison to untreated control (** *p* < 0.01 and * *p* < 0.05).

**Figure 4:**
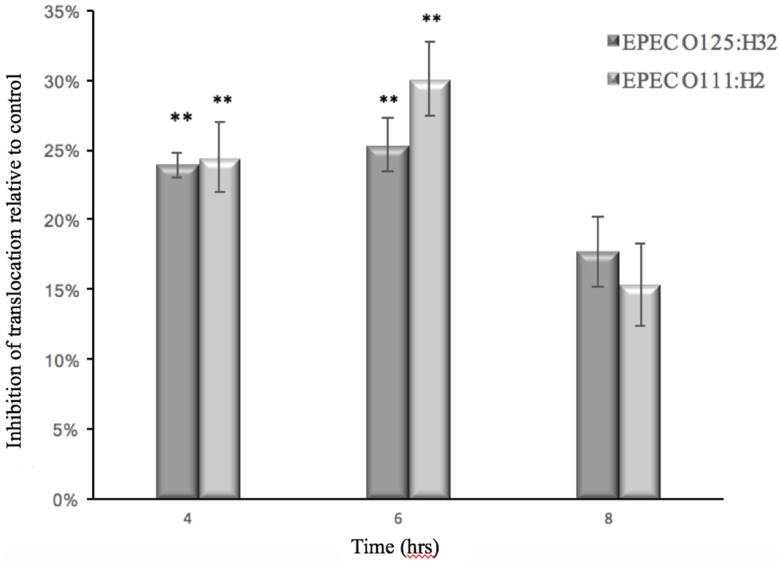
Percentage reduction of *E. coli* translocation across Caco-2 monolayers when pretreated with GMP in comparison to untreated control (** *p* < 0.01).

In order to monitor the ability of GMP to prevent barrier dysfunction, the TEER of Caco-2 cells infected with EHEC or EPEC was measured in the absence and presence of GMP. The ability of the *E. coli* strains to translocate through the cell monolayer was also measured at various time points. At 3 h post-infection with EPEC and EHEC strains, the TEER measurement of Caco-2 cells was reduced, which indicated an alteration of membrane integrity (Figure 3). Simultaneously, each of the bacteria tested—EPEC 0111:H2 and 0125:H32—penetrated the Caco-2 cell monolayer and were found in the basolateral medium 4 hours after inoculation, and bacterial numbers continued to increase over time (Figure 4). EHEC 12900 failed to penetrate the Caco-2 polarized monolayer and translocate into the basolateral media in both treated and control transwells. After 14 h, GMP’s inhibitory activity ceased, and at this point both the test and control membranes had a TEER of less than 145 Ω, which is the approximate TEER of an un-seeded well (no cell line). A decrease in TEER suggested that each strain of *E. coli* disrupted the tight junctions (TJs) of Caco-2 monolayers. GMP significantly delayed both the EHEC- and EPEC-induced decrease in TEER for 3 h (*p* < 0.01 and *p* < 0.05, respectively) (Figure 3). GMP also significantly inhibited translocation of both EPEC strains through Caco-2 monolayers into the basolateral medium for a period of 2 h (*p* < 0.01) (Figure 4).

In order for EHEC and EPEC to cause infection, the bacteria must first adhere to epithelial cells where their TTSSs, which inject TIR, are activated. After an intercellular phosphorylation process, these TIRs penetrate the host membrane from the inside-out and bind to intimin on the bacterial capsule, making a secure bond to the host cell [39]. From this position, enteropathogenic bacteria, such as EPEC and EHEC, cause attaching and effacing (A/E) lesions in the small or large intestine, which in turn causes diarrhoea [44]. Overall, the mechanism in which EHEC generate A/E lesions is similar to EPEC, but a few important differences have been identified. Unlike EPEC, EHEC do not have the plasmid that facilitates initial local adherence to epithelial cells by bundle-forming pili (BFP) that facilitate bacterial autoaggregation [45]. Using the TTSS, EHEC and EPEC insert the locus of enterocyte effacement (LEE) effector molecules into host cells. EPEC effector molecules such as EspG and EspG 2 are known to modulate paracellular permeability and tight junction disruption during infection [46,47]. The absence of such molecules from EHEC’s arsenal may explain why only EPEC translocated through the Caco-2 monolayers (Figure 4). Both EPEC and EHEC share the ability to initiate host-cell inflammation by inducing the expression of cytokines IL-8, TNF-α and IL-1β, which are pro-inflammatory cytokines that elicit the loosening and eventual destruction of TJs in human intestinal epithelial cells [10,48,49,50,51].

In agreement with the present study, it was previously shown that EHEC do not translocate through polarized Caco-2 monolayers. However, their LEE do, resulting in TNF-α and IL-8 secretion, which could cause intestinal blood-vessel destruction in humans. A number of studies [11,30,52,53] have found that GMP suppressed IL-8 production in Caco-2 cells that were challenged with other pathogens, which regulate IL-8 in a similar manner to *E. coli*. EHEC and EPEC have been shown to activate the nuclear factor-kappa B (NF-κB) pathway, which triggers IL-8 secretion in intestinal epithelial cell lines [54]. Furthermore, IL-8 production in intestinal cells increases in the presence of TNF-α, which extenuates the degradation of NF-κB inhibitor Iκ-Bα [54,55,56]. NF-κB is considered the most important nuclear transcription factor for IL-8 and TNF-α-induced tight junction degradation in human cells [57,58,59,60].

TNF-α down-regulates production of the TJ protein ZO-1, and alters the junctional distribution of ZO-1 proteins via the NF-κB pathway, which increases permeability of Caco-2 tight junctions [59]. Gong et al. [61] demonstrated that GMP inhibits the NF-κB signalling pathway in HT-29 cells when challenged with EPEC lipopolysaccharide (LPS). Therefore, GMP may promote TJ integrity by suppressing NF-κB pathway activation, which would reduce IL-8 and TNF-α-associated permeability. This could explain the delay in the *E. coli*-induced TEER decrease and EPEC translocation in GMP-treated Caco-2 monolayers, as observed in the present study.

Interestingly, Rong et al. [62] demonstrated the ability of GMP in significantly alleviating the increase of pathogenic bacteria counts in intestinal contents, intestinal morphology and acute inflammatory responses induced by *E. coli* K88 infection. Importantly, similar to the current study, the researchers found that GMP prevents intestinal barrier permeability damage induced by *E. coli* K88 infection. It was concluded that GMP supplementation in the diet protects the weaning piglets against *E. coli* infection.

The myosin light-chain kinase (MLCK) pathway is another important TJ-associated intercellular pathway triggered by EHEC and EPEC exposure [63,64]. The interaction of IL-1β with the NF-kB pathway stimulates MLCK mRNA transcription, which upregulates MLCK protein expression leading to an increase of TJ permeability in Caco-2 cell monolayers [65]. It has been demonstrated that GMP treatment suppressed IL-1β mRNA levels in rats with colitis [66]. Similarly, Monnai and Otani [67] found that GMP stimulates the release of the IL-1β antagonist known as IL-1ra in monocytes. In the current study, GMP may prevent the activation of other pathways such as MLCK, which are associated with rearrangement of TJ-associated proteins ZO-1 and occludin after infection, leading to a decrease in TEER for both *E. coli* types and subsequent paracellular movement of EPEC (Figure 3 and Figure 4). An alternative mechanism for EHEC-induced TEER reduction and inflammation, not involving LEE effector proteins, has been proposed. Ma et al [59] suggested that initial attachment of the H7 flagella of EHEC to Caco-2 cells triggers mitogen-activated protein kinase signalling pathways and the transcription factor NF-κB. This leads to the chemotaxis of IL-8 from the basolateral surface of colonic epithelial cells. It is possible that during the incubation period in the present study, GMP could be obstructing the initial attachment of EHEC’s H7 flagella to Caco-2 cell monolayers via presentation of decoy ligands, thereby lowering the number of *E. coli* available to infect, which delays the decrease in TEER.

## 4. Conclusions

GMP prevents the adhesion of various strains of EHEC and EPEC to Caco-2 and HT-29 cells, and this adhesive ability was not linked to either pathotype or cell line but appeared to be strain-specific. To the best of our knowledge, this is the first study that details GMP’s ability to maintain the structural integrity of Caco-2 TJs. Furthermore, GMP delays the paracellular movement of EPEC through the TJs of Caco-2 monolayers. Future work will focus on further establishing the strain-specificity of GMP’s anti-adhesive effects, understanding this phenomenon and investigating the mechanism of action of GMP-induced reduction of cell permeability. This study indicates that GMP has potential as a bioactive ingredient which could assist in improving the gastrointestinal health of individuals. Indeed, recently GMP has shown promise as a nutritional therapy in patients with active distal ulcerative colitis [68]. Moreover, methods for producing GMP based on its glycosylation and the effect of its glycosylation on the peptide’s techno-functional properties have been documented [69,70,71,72,73,74]. The fact that some dairy-ingredient companies have already realized the potential of GMP and are promoting this glycopeptide as a premium ingredient isolated from whey makes these findings particularly relevant.

## Figures and Tables

**Table 1:** The percentage inhibition of *E. coli* adherence to HT-29 and Caco-2 cells relative to the control.

*E. coli* strain	% Inhibition	
	HT-29	Caco-2
EHEC 12900 O157:H7	70 ***	62 ***
EPEC O111:H2	26 *	25 *
EPEC O125:H32	24 *	25 *
EHEC DAF 454	21	N/T
EHEC DPC 6055	15	N/T

EHEC = enterohemorrhagic *E. coli*; EPEC = enteropathogenic *E. coli*, N/T = not tested (*** *p* < 0.01 and * *p* < 0.05).
